# The Impact of Prehospital and Hospital Care on Clinical Outcomes in Out-of-Hospital Cardiac Arrest

**DOI:** 10.3390/jcm11226851

**Published:** 2022-11-20

**Authors:** Yotam Deri, Baruch Berzon, Debra West, Matan Machloof, Refael Strugo, Tomer Kaplan, Shelly Soffer

**Affiliations:** 1 Department of Emergency Medicine Assuta Ashdod Hospital, Ashdod 7747629, Israel; 2 Goldman Medical School, Ben-Gurion University of the Negev, Be’er Sheva 8410501, Israel; 3 Internal Medicine B, Assuta Ashdod Hospital, Ashdod 7747629, Israel; 4 Magen David Adom, Ha-Refu’a St 1, Ashdod 6021805, Israel

**Keywords:** cardiopulmonary resuscitation, out-of-hospital cardiac arrest (OHCA), return of spontaneous circulation (ROSC), automated external defibrillator, basic life support

## Abstract

**Background**: In recent years, several actions have been made to shorten the chain of survival in out-of-hospital cardiac arrest (OHCA). These include placing defibrillators in public places, training first responders, and providing dispatcher-assisted CPR (DA-CPR). In this work, we aimed to evaluate the impact of these changes on patients’ outcomes, including achieving return of spontaneous circulation (ROSC), survival to discharge, and survival with favorable neurological function. **Methods**: We retrospectively retrieved data of all calls to the national emergency medical service in Ashdod city, Israel, of individuals who underwent OHCA at the age of 18 and older between the years 2018 and 2021. Data was collected on prehospital and hospital interventions. The association between pre-hospital and hospital interventions to ROSC, survival to discharge, and neurological outcomes was evaluated. Logistic regression was used for multivariable analysis. **Results**: During the years 2018–2021, there were 1253 OHCA cases in the city of Ashdod. ROSC was achieved in 207 cases (32%), survival to discharge was attained in 48 cases (7.4%), and survival with favorable neurological function was obtained in 26 cases (4%). Factors significantly associated with good prognosis were shockable rhythm, witnessed arrest, DA-CPR, use of AED, and treatment for STEMI. All patients that failed to achieve ROSC outside of the hospital setting had a poor prognosis. **Conclusions**: This study demonstrates the prognostic role of the initial rhythm and the use of AED in OHCA. Hospital management, including STEMI documentation and catheterization, was also an important prognostication factors. Additionally, when ROSC is not achieved in the field, hospital transfer should be considered.

## 1. Introduction

Out-of-hospital cardiac arrest (OHCA) is a widespread global problem, with varying survival rates. For example, Asia reports the lowest survival-to-discharge rate (2%), followed by North America (6%), Europe (9%), and Australia (11%) [1]. In a worldwide meta-analysis, it was found that the average global return of spontaneous circulation (ROSC) rate is 29.7%, the survival-to-hospitalization rate is 22–24%, and the survival-to-discharge rate is 7.6–8.8% [2,3].

The prognosis and survival after OHCA are affected by two main processes: pre-hospital and hospital care. Pre-hospital treatment includes early detection of cardiac arrest, early initiation of cardiopulmonary resuscitation (CPR), rapid defibrillation (for a shockable rhythm), and rapid transfer to the hospital. The sooner these actions are performed, the higher the patient’s chances of survival [4]. The hospital care treatment includes continued CPR, temperature management, use of extracorporeal membrane oxygenation (ECMO), coronary catheterization, and intensive care unit (ICU) monitoring [5,6].

Due to the crucial importance of prehospital care, in recent years, several actions have been taken to shorten the emergency medical service (EMS) chain of survival and to improve the medical response in the initial minutes after cardiac arrest. These include placing defibrillators in public places, using smart phone applications, initiating the national Alert Crew Unit (first responders), and providing telephone CPR instruction to bystanders to perform CPR (dispatcher-assisted CPR).

In this work, we aimed to evaluate the above-mentioned changes in recent years on patients’ outcomes, including achieving ROSC, survival at discharge, and survival with favorable neurological function. We also examined these outcomes in the first years of the COVID-19 pandemic.

## 2. Methods

We retrospectively retrieved data of all calls to the national EMS in Ashdod, Israel, of individuals who underwent OHCA at the age of 18 and older between the years 2018 and 2021. Trauma cases were excluded. Data was collected and analyzed only in cases where a CPR attempt was performed (cases defined as do not resuscitate (DNR) or when there were signs of certain death were excluded).

An institutional review board (IRB) (IRB 0036-21-AAA) approval was granted for this retrospective study. Informed consent was waived by the IRB committee.

### 2.1. Clinical Setting

The city of Ashdod encompasses a population of 220,000 citizens. In 2017, Assuta Ashdod Hospital in the city of Ashdod was opened. A prehospital emergency service is provided in Israel by the national EMS called Magen David Adom (MDA). For each case defined as OHCA, the nearest available ambulance, first responders, and a mobile intensive care unit (MICU) are immediately dispatched.

CPR is performed in every OHCA case, except in cases where death is certain (for example, signs of decay, organ failure, etc.) or in cases where the patient has been defined as DNR (on a form signed by him or by a guardian).

In Israel, all sophomore students must by law participate in a ten-hour basic life support (BLS) course. Hence, the general population is familiar with the chest compression procedure and is aware of the urgency in attaining an automated external defibrillator (AED) during cardiac arrest. According to the law in Israel, an AED must be available in all public places that are meant to contain more than 500 people, such as central bus stations, and in sensitive locations such as elderly homes, gyms, and public pools. In recent years, in the city of Ashdod, an effort has been made to place even more AED devices than required by the law. For example, the EMS service has added 71 AED stations.

### 2.2. Prehospital Care Data Collection

Every case of OHCA that underwent CPR in the field is documented in the MDA standardized medical record by the team leader in the field. Actions that took place prior to the arrival of the team (such as dispatcher-assisted CPR) are documented by the person on duty at the MDA call center.

The data collected from the MDA database includes: ID number, date, age, gender, whether a dispatcher-assisted CPR was attempted, OHCA occurrence location (home or public place), moment of collapse (observed by bystanders, observed by medical professionals, not observed, or unknown), whether BLS was performed prior to the arrival of MICU (not performed at all, performed by bystanders who were not medical personnel, performed by medical professionals), whether there was use of an AED device (did not use at all, shock was given, used but shock was not given, unknown), who used the AED (bystanders, first responders, EMS, unknown, not recorded), and the first rhythm recorded by MICU: ventricular fibrillation (VF)\ventricular tachycardia (VT), pulseless electrical activity (PEA)\asystole, normal sinus rhythm (NSR)\atrial fibrillation (AFIB)\PACE, and ROSC (whether pulse was returned or not).

### 2.3. Hospital Care Data Collection

Patients who underwent CPR and who were not declared dead in the field were transferred to Assuta Ashdod Hospital. Most of the patients were transferred to the shock room at the hospital, and a small number were transferred directly to the cardiac catheterization laboratory. The information was recorded in the hospital electronic medical records (EMRs). The following data were collected: cardiovascular (CV) risk factors, patient’s functional status before the OHCA, ST-elevation myocardial infarction (STEMI) recorded on hospital ECG, cardiac catheterization, mortality, and patient’s neurological function at discharge according to the cerebral performance category (CPC) index, where favorable function is CPC 1–2 and unfavorable function is CPC 3–4 (CPC 1: good cerebral performance with a minor neurologic or psychologic deficit; CPC 2: moderate cerebral disability where the patient has sufficient cerebral function for independent activities of daily life; CPC 3: severe cerebral disability where the patient is dependent on others for daily support due to impaired brain function; CPC 4: coma or vegetative state [7]).

### 2.4. Primary End Point

To evaluate the association between prehospital and hospital interventions with ROSC, survival at discharge and neurological outcome.

### 2.5. Secondary Endpoint

To examine the rates of ROSC, survival at discharge, and neurological outcome during the first year of the COVID-19 pandemic.

### 2.6. Statistical Analysis

Categorical variables were summarized as a frequency and percentage. Continuous variables were evaluated for normal distributions using the Kolmogorov–Smirnov test. Since all continuous variables were skewed, they were reported as a median and interquartile rate. The Mann–Whitney test was applied to compare continuous variables while the Chi-square test and Fisher-exact test were used to compare categorical variables. Logistic regression was used for multivariable analysis. The model was adjusted for sex and gender. Adjusted odds ratios (aORs), 95% confidence intervals (CIs), and *p*-values were calculated for the models’ variables. All statistical tests were two-sided, and *p* < 0.05 was considered as statistically significant. NCSS software was used for all statistical analysis (NCSS 2022 Statistical Software (2022). NCSS, LLC., Kaysville, UT, USA).

## 3. Results

**Figure 1:**
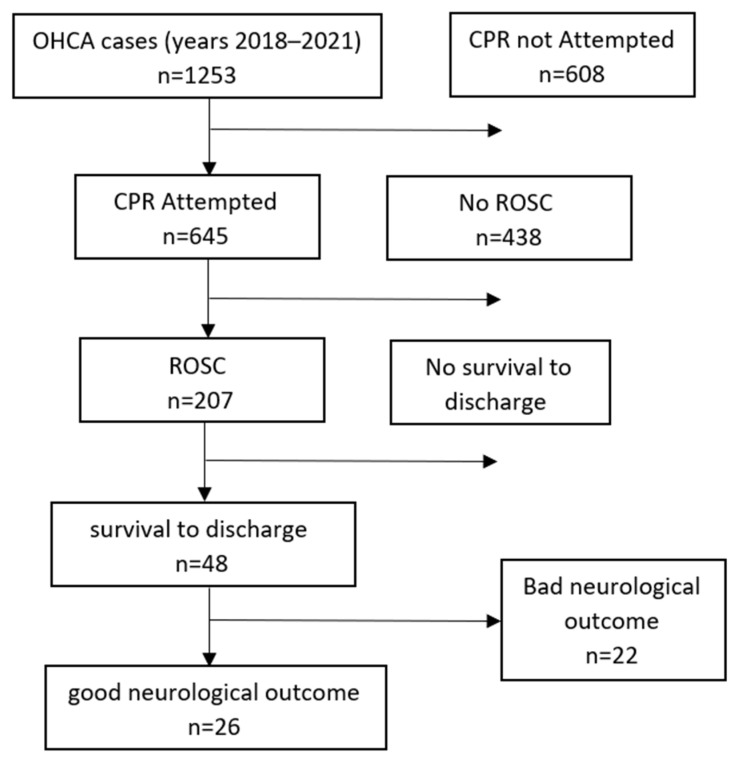
Study flow chart.

During the years 2018–2021, there were 1253 OHCA cases in the city of Ashdod (Figure 1).

Of these, CPR was performed in 645 cases. ROSC was achieved in 207 cases (32%), survival to discharge was attained in 48 cases (7.4%), and survival with favorable neurological function was obtained in 26 cases (4%).

In 46 cases, ROSC was not achieved in the field and yet the patient was transferred to the hospital. In only three of these cases, ROSC was attained in the hospital and only one of them achieved survival to discharge with an unfavorable neurological outcome.

### 3.1. Pre-Hospital and Hospital Care

Of the 645 cases who received CPR, 118 occurred in a public location (18.3%) and 438 were witnessed (67.9%). BLS before MICU was performed by bystanders in 165 cases (25.5%) and by professionals in 333 cases (51.6%). AED was used in 284 cases (44%), but it was used by bystanders in only 10 cases (1.5%). DA-CPR was performed in 233 of the cases (36.1%). Of the 207 patients that achieved ROSC, 52 patients had documented STEMI on ECG (25.1%) and 40 patients went through a cardiac catheterization procedure (19.3%).

### 3.2. ROSC

Table 1 demonstrates the characteristics of the group in which ROSC was achieved compared to the group in which ROSC was not achieved. Patients with a successful ROSC were significantly younger (75 years old (IQR 66–85) vs. 80 years old (IQR 70–88), PV = 0.004). A higher ROSC rate was obtained among those who had the OHCA in a public place compared to those who had the OHCA at home (45.7% vs. 29%; PV < 0.001). A higher ROSC rate was achieved if the moment of collapse was observed by the bystanders and EMS compared to cases where the moment of collapse was not observed at all (41.0% and 40.3% vs. 12.3%; PV < 0.001). The ROSC rate was higher in cases where initial BLS before MICU arrival was performed by bystanders compared to cases in which it was performed by professionals (38.1% vs. 27.6%; PV < 0.001). In cases where DA-CPR was performed, there were better ROSC rates compared to cases where it was not performed (37.7% vs. 24.8%, PV < 0.001).

A higher ROSC rate was observed in cases where AED was used and a shock was given compared to cases where it was not used (50% vs. 35.8%; PV = 0.03). If the initial rhythm recorded in MICU was VF/VT, there was a higher chance of ROSC compared to PEA/Asystole (59.0% vs. 28.8%; PV < 0.001).

In the logistic regression model adjusted for age and gender, factors significantly associated with ROSC were location (home vs. public OR 0.226, CI 0.369–0.957, PV = 0.032), witnessed arrest (unwitnessed vs. bystanders OR 0.226, CI 0.136–0.374, PV < 0.001), initial rhythm (PEA/asystole vs. VF\VT OR 0.5, PV 0.0434, CI 0.257–0.980), and DA-CPR (underwent DA-CPR vs. did not undergo DA-CPR OD 1.633, CI 1.007–2.650, PV = 0.046) (Table 2).

### 3.3. Survival to Discharge

Table 3 shows the characteristics of all cases referred to Assuta Ashdod Hospital (total of 215), comparing between cases in which survived to discharge was achieved and cases in which survival to discharge was not attained. Patients who survived at discharge were younger (64 (52.25–75.5) vs. 77 (67–86), PV < 0.001), were primarily men (29.6% vs. 12.2%, PV < 0.001), had less CV comorbidities (18.8% vs. 50%, PV < 0.001), and had a better functional status (35.9% vs. 9.8%, PV < 0.001). The survival-to-discharge rate was higher in cases in which BLS was performed by bystanders compared to cases in which BLS was not performed before the MICU arrival (30.6% vs. 12.7%; PV < 0.001). The survival-to-discharge rate was higher in cases where an AED was used and shock was given compared to cases where AED was not used or AED was used but shock was not given (51.4% vs. 19.6% vs. 13.3%; PV < 0.001). A higher survival-to-discharge rate was observed when AED was used by bystanders compared to a first responder and EMS (83.3% vs. 40.0% and 15.4%; PV < 0.001) and compared to cases in which it was not used at all (PV < 0.001).

The survival-to-discharge rate was higher in cases where the initial rhythm was VF\VT compared to PEA\asystole (64.7% vs. 12.4%, PV < 0.001). Additionally, the survival to discharge was higher if the initial rhythm was NSR\AFIB\PACE compared to PEA\asystole (41.7% vs. 12.4%; PV < 0.001).

In cases where STEMI was documented, the chances of survival to discharge were higher compared to cases where STEMI was documented (51.9% vs. 12.7%; PvC < 0.001). Furthermore, in cases where cardiac catheterization was performed, there was a higher chance of survival to discharge (70% vs. 11.4%; PV < 0.001).

In the logistic regression model adjusted for age and gender, factors significantly associated with survival to discharge were AED use (use with shock delivery vs. no use OR 2.629, CI 1.109–6.229, PV 0.028), AED user (use by bystander vs. no use OR 12, CI 1.24–116.2, PV 0.03 and use by first responder vs. no use OR 2.54, CI 1.07–6, PV = 0.03), initial rhythm (PEA/asystole vs. VF\VT OR 0.114, CI 0.046–0.280, PV < 0.001), documented STEMI in ECG (no STEMI vs. STEMI OR 0.171, CI 0.074–0.390, PV < 0.001), cardiac catheterization (underwent catheterization vs. did not undergo catheterization OR 12.952, CI 5.426–30.918, PV < 0.001), and functional status before the OHCA (high functional status vs. dependent functional status OR 2.869, CI 1.228–6.702, PV = 0.014) (Table 4).

### 3.4. Neurological Outcome

Table 5 shows a comparison between cases in which a favorable neurological outcome was achieved and cases in which an unfavorable neurological outcome was attained. Patients who achieved a better neurological outcome were younger (58.5, IQR 51.25–72 vs. 76, IQR 65.5–86; PV < 0.001), were primarily men (16% vs. 6.7%; PV = 0.003), had less CV comorbidities (45.8% vs. 7.9%; PV < 0.001), and had a better functional status (25.2% vs 0%; PV < 0.001).

Those who underwent OHCA in a public place achieved a better neurological outcome compared to those who underwent OHCA at home (18.6% vs. 9.0%; PV = 0.04). In cases where BLS was performed by bystanders, there was a higher chance of a favorable neurological outcome compared to cases where BLS was not performed before the MICU arrival (12.9% vs. 2.82%; PV = 0.04). In cases where BLS was performed by a professional, there was a higher rate of a favorable neurological outcome compared to cases where BLS was not performed before the MICU arrival (19.5% vs. 2.82%; PV < 0.001).

When AED was used and a shock was given, there was a better neurological outcome compared to cases where it was not used or was used but shock was not given (35.1% vs. 7.8% and 6.7%, PV < 0.001). When AED was used by bystanders, there was the highest chance of a good neurological outcome compared to cases where it was used by a first responder, EMS, or not used at all (83.3% vs. 25%, 7.7%, and 6%; PV < 0.001).

When the initial rhythm recorded by MICU was shockable (VF/VT), there was a higher rate of a favorable neurological outcome compared to cases where the initial rhythm recorded was not shockable (PEA/asystole) (50% vs. 3.0%; PV < 0.001).

When STEMI was documented, there was a higher rate of a favorable neurological outcome compared to cases where STEMI was not documented (30.8% vs. 5.9%; PV < 0.001). When cardiac catheterization was performed there was a better neurological outcome (50% vs. 3.4%; PV < 0.001).

In the logistic regression model adjusted for age and gender, factors significantly associated with a favorable neurological outcome were AED user (use by bystander vs. no use OR 44.614, CI 4.154–479.158, PV = 0.001 and use by first responder vs. no use OR 4.647, CI 1.476–14.626, PV = 0.008, respectively), initial rhythm (PEA/asystole vs. VF\VT OR 0.035, CI 0.009–0.127, PV < 0.001), documented STEMI in ECG (no STEMI vs. STEMI OR 0.177, CI 0.061–0.510, PV = 0.001), cardiac catheterization (underwent catheterization vs. did not undergo catheterization OR 23.120, CI 7.335–72.878, PV < 0.001), CV comorbidities (no CV risk factors vs. CV risk factors OR 4.956, CI 1.620–15.159, PV = 0.005), and functional status before the OHCA (high functional status vs. dependent functional status OR > 10,000, CI 173.918–10,000+, PV < 0.001) (Table 6).

### 3.5. First Year of the COVID-19 Pandemic

In the first year of the COVID-19 pandemic, no differences were found in all three outcomes compared to the examined years before the onset of the pandemic (Table 7).

## 4. Discussion

In this study, based on hospital and EMS data, we evaluated the association between prehospital care and hospital care on patients’ outcome. We demonstrated the value of the initial rhythm and the importance of AED in OHCA on all three outcomes. Hospital management, including STEMI documentation and catheterization, was also an important prognostication factor.

Similar to the results demonstrated in a meta-analysis [2,3], in our study, the ROSC rate was about one-third of the cases in which CPR was performed. The survival-to-discharge rate (7.4%) was also similar to those presented in the world (7.1–8.8%). This emphasizes that ROSC is only one index for successful resuscitation. A high ROSC rate does not necessarily indicate a high survival rate or favorable neurological function.

Patients who achieved ROSC, survival to discharge, and a favorable neurological outcome were significantly younger. A similar trend was presented in the study of Einav et al. [8]. Men had a better chance of achieving survival to discharge (PV = 0.002) and favorable neurological function (PV = 0.038) than women. It was suggested that this gender difference is related to the low rate of shockable rhythm among women [9,10].

OHCA in a public place was found to be a factor that significantly increased the rate of ROSC. A similar trend, although not significant, was also demonstrated for the effect of OHCA in a public place on survival to discharge and neurological function outcomes. In accordance with these findings, in cases where OHCA was unwitnessed, a negative effect on the chance of ROSC was found. This is not surprising since in crowded public places (such as educational institutions, nursing homes, gyms), there is an AED device available. Similar findings were also found in previous studies [11,12].

AED use with shock administration showed a significant improvement in all final outcomes. Shock administration by AED results in a decreased response time and a reduced chain of survival time. Additionally, as shown before [13,14,15], we demonstrated that when a shockable rhythm is documented, a better prognosis is achieved, with an improvement in all three final outcomes. This may be due to the fact that the shockable rhythm is often early in the OHCA process [16]. It was also found, similarly to previous studies [11,13,17], that the use of AED by bystanders has the best success rates, followed by the use of AED by first responders, and finally by the EMS team. In cases where it is a shockable rhythm, the sooner a defibrillator is connected and a shock is given, the higher the patient’s chances of survival. Compared to the performance of BLS by bystanders, the use of AED demonstrated a higher significant improvement in prognosis. Prior meta-analysis has emphasized the importance of both chest compressions and the use of AED [18]. Our research demonstrates a greater positive effect for defibrillation. However, the use of defibrillation among bystanders was very limited. This further emphasizes the crucial need for a greater number of AED devices and for the expansion of awareness and training of bystanders. Mandatory training, which is currently available only for sophomore students, should be expanded to include a wider number of settings.

As previously demonstrated [19,20], in cases where STEMI was documented on ECG and the patient was catheterized, the prognosis was better compared to those whose STEMI was not observed and therefore were not catheterized.

In this study, there were 46 patients who were transferred to the hospital even though they did not achieve ROSC in the field, and only one of them survived to discharge but without a good neurological outcome. Hence, there was no apparent clinical benefit in hospital transfer for the patients who did not achieve ROSC in the field. In a large study conducted in Japan, it was found that only 0.2% of the patients who did not achieve ROSC and were referred to the emergency room (despite meeting the criteria for termination of resuscitation according to the universal termination of resuscitation rule) were discharged with favorable neurological function [21]. In the event that CPR in the field did not achieve ROSC, transport to a hospital should be seriously considered given the large resources invested in these patients and the poor prognosis. The decision to forgo hospital transfer can prevent unnecessary patient and family suffering and can allow for efficient resource reallocation.

This study did not demonstrate a change in the prognosis (ROSC, survival to discharge, and neurological function) in the first year of the COVID-19 pandemic in Israel (March 2020–March 2021) compared to the previous years. However, recent meta-analyses have shown a worse prognosis during the pandemic, which has been attributed to lower rates of bystander defibrillation use, longer time of EMS arrival, and a longer chain of survival [22,23,24].

Our study has several limitations. This was an observational retrospective analysis. Additionally, since Assuta hospital is a new hospital, this study examined the years 2018–2021, and a comparison could not be made to prior years in the Ashdod city. The comparison was thus carried out using global data extracted from the relevant literature. A further limitation relates to the lack of an immediately available ECMO team in Assuta Ashdod hospital. The fact that extracorporeal cardiopulmonary resuscitation (eCPR) was not utilized may have influenced the results. Furthermore, in Israel, there is a cultural and religious tendency to perform resuscitation among cases of terminally ill patients, in contrast to the acceptable and typical norm in other countries.

## 5. Conclusions

A large gap was observed between the cases in which ROSC was achieved and the cases in which a good neurological outcome was attained, despite the rapid arrival of EMS and overall good prehospital care. A larger number of AEDs in the public domain and more extensive training of first responders could reduce this gap. Additionally, ethical and legal considerations should be taken into account in order to reduce the initiation of CPR in cases where the patient’s basic condition is poor, for example by electronic registration of patients defined as DNR. It is also important to note that ROSC is only one index among other indices, including survival to discharge and neurological function. When building a treatment protocol and when approaching CPR, it is recommended to consider these indicators as well. When ROSC is not achieved in the field, the transfer of patients to the hospital has no apparent benefit and therefore should not be undertaken.

## Figures and Tables

**Table 1:** Crude ROSC outcome according to patients’ characteristics and prehospital and hospital care.

Characteristic		No ROSC (438)	ROSC (*n* = 207)	PV
**Gender**	Male	229 (65.6%)	120 (34.4%)	0.176
	Female	209 (70.6%)	87 (29.4%)	
**Age (years)**	median (IQR)	80 (70–88)	75 (66–85)	**0.004**
**Location**	Public	64 (54.3%)	54 (45.7%)	**<0.001**
	Home	374 (71.0%%)	153 (29.0%)	
**Witnessed status**	Bystanders	191 (59.0%)	133 (41.0%)	**<0.001**
	EMS	68 (59.7%)	46 (40.3%)	
	Unwitnessed	164 (87.7%)	23 (12.3%)	
	Unknown	15 (75%)	5 (25%)	
**BLS before MICU**	No	96 (65.3%)	51 (34.7%)	**0.031**
	By bystanders	101 (61.2%)	64 (38.8%)	
	By professionals	241 (72.3%)	92 (27.6%)	
**AED use**	No	158 (64.2%)	88 (35.8%)	**<0.001**
	Yes and shock	34 (50.0%)	34 (50.0%)	
	Yes and no shock	163 (75.5%)	53 (24.5%)	
	Unknown	83 (72.2%)	32 (27.8%)	
**AED user**	Bystanders	4 (40.0%)	6 (60.0%)	
	On-calls	91 (69.0%)	41 (31.0%)	0.136
	EMS	98 (69.0%)	44 (31.0%)	
	Unknown	89 (74.2%)	31 (25.8%)	
	Didn’t use	156 (64.7%)	85 (35.3%)	
**First documented rhythm by MICU**	VT/VF	23 (41.0%)	33 (59.0%)	**<0.001**
	PEA/Asystole	401 (71.2%)	162 (28.8%)	
	All others	14 (53.9%)	12 (46.1%)	
**Minute to arrival**	median (IQR)	6 (4–8)	6 (4–8)	0.836
**DA-CPR**	didn’t try (1)	230 (75.2%)	76 (24.8%)	**<0.001**
	tried but didn’t do (2)	8 (80%)	2 (20%)	
	done (3)	145 (62.2%)	88 (37.8%)	
	Arrest witnessed by EMS (4)	55 (57.3%)	41 (42.7%)	

All percentages were calculated from the total for that column. EMSs, emergency medical services; MICU, mobile intensive care unit; VF, ventricular fibrillation; VT, ventricular tachycardia; PEA, pulseless electrical activity; CPR, cardiopulmonary resuscitation; DA-CPR, dispatcher-assisted CPR; PV: P Value; BLS—basic life support; IQR: Interquartile range; ROSC: return of spontaneous circulation

**Table 2:** Adjusted ROSC outcome according to patients’ characteristics and prehospital and hospital care.

Characteristic		ROSC		
		Adjusted OR	CI 95%	PV
**Gender**	Male (reference)			
	Female	1.084	0.743–1.582	0.674
**Location**	Public (reference)			
	Home	0.594	0.369–0.957	**0.032**
**Witnessed status**	Bystanders (reference)			
	EMS	0.562	0.214–1.480.244	0.244
	Unwitnessed	0.226	0.369–0.957	**0.032**
	Unknown	0.512	0.173–1.510	0.225
**BLS before MICU**	No (reference)			
	By bystanders	1.102	0.527–2.301	0.795
	By professionals	0.903	0.469–1.738	0.76
**AED Use**	No (reference)			
	Yes and shock	1.09676	0.530–2.265	0.803
	Yes and no shock	0.88338	0.494–1.577	0.675
	Unknown	0.96074	0.506–1.823	0.902
**First documented rhythm by MICU**	VT/VF (reference)			
	PEA/asystula	0.5	0.257–0.980	**0.043**
	All others	0.745	0.271–2.044	0.568
**DA-CPR**	Did not try (reference)			
	Tried but didn’t do	0.758	0.148–3.867	0.739
	Done	1.63	1.007–2.650	**0.046**
	Collapse by EMS	2.141	0.801–5.727	0.129

OR, odds ratio; CI, confidence interval; EMSs, emergency medical services; MICU, mobile intensive care unit; VF, ventricular fibrillation; VT, ventricular tachycardia; PEA, pulseless electrical activity; CPR, cardiopulmonary resuscitation; DA-CPR, dispatcher-assisted CPR. Adjustable for age, gender, location, witnessed status, BLS before MICU, AED use, first rhythm by MICU, DA-CPR; PV: P Value; BLS: basic life support; ROSC: return of spontaneous circulation

**Table 3:** Crude survival to discharge outcome according to patients’ characteristics and prehospital and hospital care.

Characteristic		Did not Survive to Discharge (*n* = 167)	Survived to Discharge (*n* = 48)	PV
**Gender**	Male	88 (70.4%)	37 (29.6%)	**0.0025**
	Female	79 (87.8%)	11 (12.2%)	
**Age (years)**	median (IQR)	77 (67–86)	64 (52.25–75.5)	**<0.0001**
**Location**	Public	49 (70.0%)	21 (30.0%)	0.0604
	Home	118 (81.4%)	27 (18.6%)	
**Witnessed status**	Bystanders	81 (72.3%)	31 (27.7%)	0.1311
	EMS	59 (80.8%)	14 (19.2%)	
	Unwitnessed	23 (92.0%)	2 (8.0%)	
	Unknown	4 (80.0%)	1 (20.0%)	
**BLS before MICU**	No	62 (87.3%)	9 (12.7%)	**0.039**
	By bystanders	43 (69.4%)	19 (30.6%)	
	By professionals	62 (75.6%)	20 (24.4%)	
**AED Use**	No	82 (80.4%)	20 (19.6%)	**<0.001**
	Yes and shock	18 (48.6%)	19 (51.4%)	
	Yes and no shock	39 (86.7%)	6 (13.3%)	
	Unknown	28 (90.3%)	3 (9.7%)	
**AED user**	Bystanders	1 (16.7%)	5 (83.3%)	**<0.001**
	On-calls	24 (60.0%)	16 (40.0%)	
	EMS	33 (84.6%)	6 (15.4%)	
	Unknown	27 (90.0%)	3 (10.0%)	
	Didn’t use	82 (82.0%)	18 (18.0%)	
**First documented rhythm by MICU**	VT/VF	12 (35.3%)	22 (64.7%)	**<0.001**
	PEA/asystula	148 (87.6%)	21 (12.4%)	
	All others	7 (58.3%)	5 (41.7%)	
**Minutes to arrival**	median (IQR)	6 (4–9)	5.5 (4–7.75)	0.23682
**DA-CPR**	Did not try	53 (81.5%)	12 (18.5%)	0.5803
	Tried but didn’t do	2 (100%)	0 (0%)	
	Done	59 (72.8%)	22 (27.2%)	
	Collapse by EMS	53 (79.1%)	14 (20.9%)	
**STEMI**	Yes	25 (48.1%)	27 (51.9%)	**<0.001**
	No	89 (87.3%)	13 (12.7%)	
	No ECG	53 (86.9%)	8 (13.1%)	
**Catheterization**	NO	155 (88.6%)	20 (11.4%)	**<0.001**
	Yes	12 (30.0%)	28 (70.0%)	
**CV Comorbidities**	No	12 (50%)	12 (50%)	**<0.001**
	Yes	155 (81.2%)	36 (18.8%)	
**Basic function**	Dependent	101 (90.2%)	11 (9.8%)	**<0.001**
	Independent	66 (64.1%)	37 (35.9%)	

All percentages are calculated from the total for that column. EMSs, emergency medical services; MICU, mobile intensive care unit; VF, ventricular fibrillation; VT, ventricular tachycardia; PEA, pulseless electrical activity; CPR, cardiopulmonary resuscitation; DA-CPR, dispatcher-assisted CPR. STEMI, ST elevation myocardial infarction; CV, cardiovascular. PV: P Value; BLS: basic life support.

**Table 4:** Adjusted survival to discharge outcome according to patients’ characteristics and prehospital and hospital care.

Characteristic		Survival to Discharge		
		Adjusted OR	CI 95%	PV
**BLS before MICU**	No (reference)			
	By bystanders	1.916	0.749–4.9	0.17423
	By professionals	1.633	0.656–1.063	0.29131
**AED Use**	No (reference)			
	Yes and shock	2.629	1.109–6.229	**0.028**
	Yes and no shock	0.569	0.203–1.596	0.284
	Unknown	0.347	0.091–1.323	0.121
**AED User**	Bystanders	12	1.24–116.2	**0.03**
	On-calls	2.54	1.07–6	**0.03**
	EMS	0.059	02–1.71	0.33
	Unknown	0.387	0.09–1.5	0.17
	Did not use (reference)			
**First documented rhythm by MICU**	VT/VF (reference)			
	PEA/asystole	0.114	0.046–0.280	**<0.001**
	All others	0.499	0.125–1.994	0.325
**STEMI**	Yes (reference)			
	No	0.171	0.074–0.390	**<0.001**
	No ECG	0.2	0.076–0.523	**0.001**
**Catheterization**	No (reference)			
	Yes	12.95	5.426–30.918	**<0.001**
**CV comorbidities**	Yes (reference)			
	No	2.073	0.757–5.678	0.155
**Basic function**	Dependent (reference)			
	Independent	2.869	1.228–6.702	**0.014**

OR, odds ratio; CI, confidence interval; EMSs, emergency medical services; MICU, mobile intensive care unit; VF, ventricular fibrillation; VT, ventricular tachycardia; PEA, pulseless electrical activity; CPR, cardiopulmonary resuscitation; DA-CPR, dispatcher-assisted CPR. Adjustable for age and gender. PV: P Value; BLS: basic life support; STEMI: ST-elevation myocardial infarction; CV; cardiovascular; ECG: electrocardiogram.

**Table 5:** Crude neurological outcome according to patients’ characteristics and prehospital and hospital care.

Characteristic		Non-Favorable Neurological Outcome (*n* = 189)	Favorable Neurological Outcome (*n* = 26)	PV
**Gender**	Male	105 (84.0%)	20 (16.0%)	**0.038**
	Female	84 (93.3%)	6 (6.7%)	
**Age (years)**	median (IQR)	76 (65.5–86)	58.5 (51.25–72)	**<0.001**
**Location**	Public	57 (81.4%)	13 (18.6%)	**0.042**
	Home	132 (91.0%)	13 (9.0%)	
**Witnessed status**	Bystanders	95 (84.8%)	17 (15.2%)	0.436
	EMS	65 (89.0%)	8 (11.0%)	
	Unwitnessed	24 (96.0%)	1 (4.0%)	
	Unknown	5 (100%)	0 (0%)	
**BLS before MICU**	No	69 (97.2%)	2 (2.8%)	**0.006**
	By bystanders	54 (87.1%)	8 (12.9%)	
	By professionals	66 (80.5%)	16 (19.5%)	
**AED Use**	No	94 (92.2%)	8 (7.8%)	**<0.001**
	Yes and shock	24 (64.9%)	13 (35.1%)	
	Yes and no shock	42 (93.3%)	3 (6.7%)	
	Unknown	29 (93.5%)	2 (6.5%)	
**AED user**	Bystanders	1 (16.7%)	5 (83.3%)	**<0.001**
	On-calls	30 (75%)	10 (25%)	
	EMS	36 (92.3%)	3 (7.7%)	
	Unknown	28 (93.3%)	2 (6.7%)	
	Did not use	94 (94.0%)	6 (6.0%)	
**First documented rhythm by MICU**	VT/VF	17 (50%)	17 (50%)	**<0.001**
	PEA/asystula	164 (97.0)	5 (3.0%)	
	All others	8 (66.7%)	4 (33.3%)	
**Minutes to arrival**	median (IQR)	6 (4–9)	6 (4–7.25)	0.536
**DA-CPR**	Did not try	58 (9.2%)	7 (10.8%)	0.922
	Tried but did not do	2 (100%)	0 (0%)	
	Done	70 (86.4%)	11 (13.6%)	
	Collapse by EMS	59 (88.1%)	8 (11.9%)	
**STEMI**	Yes	36 (69.2%)	16 (30.8%)	**<0.001**
	No	96 (94.1%)	6 (5.9%)	
	No ECG	57 (93.4%)	4 (6.6%)	
**Catheterization**	NO	169 (96.6%)	6 (3.4%)	**<0.001**
	Yes	20 (50.0%)	20 (50.0%)	
**CV Comorbidities**	No	176 (92.1%)	15 (7.9%)	**<0.001**
	Yes	13 (54.2%)	11 (45.8%)	
**Basic function**	Dependent	112 (100%)	0 (0%)	**<0.001**
	Independent	77 (74.8%)	26 (25.2%)	

All percentages were calculated from the total for that column. EMSs, emergency medical services; MICU, mobile intensive care unit; VF, ventricular fibrillation; VT, ventricular tachycardia; PEA, pulseless electrical activity; CPR, cardiopulmonary resuscitation; DA-CPR, dispatcher-assisted CPR. STEMI, ST elevation myocardial infarction; CV, cardiovascular.

**Table 6:** Adjusted neurological outcome according to patients’ characteristics and prehospital and hospital care.

Characteristic		Good Neurological Outcome		
		Adjusted OR	CI 95%	PV
**Location**	Public (reference)			
	Home	0.674	0.264–1.585	0.341
**BLS before MICU**	No (reference)			
	By bystanders	2.9	0.563–14.939	0.202
	By professionals	6.026	1.283–28.3	**0.022**
**AED Use**	No (reference)			
	Yes and shock	3.96	1.389–11.289	**0.01**
	Yes and no shock	0.732	0.175–3.059	0.669
	Unknown	0.609	0.115–3.211	0.559
**AED User**	Bystanders	44.614	4.154–479.15	**0.001**
	On-calls	4.64	1.476–14.626	**0.008**
	EMS	0.93	0.209–4.133	0.924
	Unknown	0.833	0.149–4.647	0.835
	Did not use (reference)			
**First documented rhythm by MICU**	VT/VF (reference)			
	PEA/asystole	0.035	0.009–0.127	**<0.001**
	All others	0.612	0.141–2.66	0.513
**STEMI**	Yes (reference)			
	No	0.177	0.061–0.51	**0.001**
	No ECG	0.233	0.067–0.801	**0.02**
**Catheterization**	No (reference)			
	Yes	23.12	7.33–72.87	**<0.001**
**CV comorbidities**	Yes (reference)			
	No	4.956	1.62–15.159	**0.005**
**Basic function**	Dependent (reference)			
	Independent	>10,000	173.91–10,000+	**0.001**

OR, odds ratio; CI, confidence interval; EMSs, emergency medical services; MICU, mobile intensive care unit; VF, ventricular fibrillation; VT, ventricular tachycardia; PEA, pulseless electrical activity; CPR, cardiopulmonary resuscitation; DA-CPR, dispatcher-assisted CPR. Adjusted for age and gender. PV: P Value; BLS: basic life support; STEMI: ST-elevation myocardial infarction; CV: cardiovascular; ECG: electrocardiogram.

**Table 7:** Comparison between the first year of the COVID-19 pandemic and the examined years before in all three outcomes.

		COVID-19 Period	Non-COVID-19 Period	PV
**ROSC**	No	214 (67.72%)	224 (68.09%)	0.92
	Yes	102 (32.28%)	105 (31.91%)	
**Survival to discharge**	No	81 (76.41%)	88 (79.27%)	0.61
	Yes	25 (23.58%)	23 (20.72%)	
**Neurological outcome**	Favorable	93 (87.73%)	98 (88.28%)	0.9
	Unfavorable	13 (12.26%)	13 (11.71%)	

PV: P Value; ROSC: return of spontaneous circulation.

## Data Availability

The data presented in this study are available on request from the corresponding author. The data are not publicly available due to privacy.
